# Environmental Constraints for Intelligent Internet of Deep-Sea/Underwater Things Relying on Enterprise Architecture Approach

**DOI:** 10.3390/s24082433

**Published:** 2024-04-10

**Authors:** Charbel Geryes Aoun, Noura Mansour, Fadi Dornaika, Loic Lagadec

**Affiliations:** 1 ICAM (Institut Catholique d’Arts et Metiers) School of Engineering, Toulouse Campus, 75 av. de Grande Bretagne, CS 97615, CEDEX 3, 31076 Toulouse, France; 2 Lab-STICC, CNRS UMR 6285, ENSTA (Ecole Nationale Superieure de Techniques Avancees), 29806 Brest, France; loic.lagadec@ensta-bretagne.fr; 3 Department of Computer Science and Artificial Intelligence, University of the Basque Country UPV/EHU, 20018 San Sebastian, Spain; fadi.dornaika@ehu.eus; 4 IKERBASQUE, Basque Foundation for Science, 48009 Bilbao, Spain

**Keywords:** IIoUT, Smart Sensors, Smart Fusion Servers, Domain-Specific Modeling Languages, ArchiMate, Enterprise Architecture, Marine Observatories, NS-3, IMS

## Abstract

Through the use of Underwater Smart Sensor Networks (USSNs), Marine Observatories (MOs) provide continuous ocean monitoring. Deployed sensors may not perform as intended due to the heterogeneity of USSN devices’ hardware and software when combined with the Internet. Hence, USSNs are regarded as complex distributed systems. As such, USSN designers will encounter challenges throughout the design phase related to time, complexity, sharing diverse domain experiences (viewpoints), and ensuring optimal performance for the deployed USSNs. Accordingly, during the USSN development and deployment phases, a few Underwater Environmental Constraints (UECs) should be taken into account. These constraints may include the salinity level and the operational depth of every physical component (sensor, server, etc.) that will be utilized throughout the duration of the USSN information systems’ development and implementation. To this end, in this article we present how we integrated an Artificial Intelligence (AI) Database, an extended ArchiMO meta-model, and a design tool into our previously proposed Enterprise Architecture Framework. This addition proposes adding new Underwater Environmental Constraints (UECs) to the AI Database, which is accessed by USSN designers when they define models, with the goal of simplifying the USSN design activity. This serves as the basis for generating a new version of our ArchiMO design tool that includes the UECs. To illustrate our proposal, we use the newly generated ArchiMO to create a model in the MO domain. Furthermore, we use our self-developed domain-specific model compiler to produce the relevant simulation code. Throughout the design phase, our approach contributes to the handling and controling of the uncertainties and variances of the provided quality of service that may occur during the performance of the USSNs, as well as reducing the design activity’s complexity and time. It provides a way to share the different viewpoints of the designers in the domain of USSNs.

## 1. Introduction

The Intelligent Internet of Underwater Things (IIoUT) is proposed for sensing, collecting, and storing underwater information [1]. IIoUT is an application based on Underwater Smart Sensor Networks (USSNs) since it enhances deep sea monitoring; the tracking of various aquatic creatures; and ocean monitoring and observation systems, which are based on acoustic communication [2,3]. Logically, USSNs operate in the same manner as Smart Sensor Networks (SSN) that are not submerged in water, providing the same services, such as deep sea/underwater monitoring. However, the unreliable transmission medium, erratic radio signals, constrained bandwidth, low transmission rate, sluggish propagation speed, inborn noise, node mobility, lesser resources, and restricted battery capacity present obstacles for USSNs. Channel modeling, optimal routing, security, privacy, communication overhead, congestion control, packet error rate, packet latency, energy consumption, and other problems are brought on by these difficulties [4,5]. By making such information systems’ design phase less complex, this article seeks to address some of the aforementioned issues, such as the complexity of modeling USSNs.

Thus, we can conclude that the USSN deployment phase is different from the non-underwater phase since during this phase the experts have to take into account certain underwater environmental constraints. In general, an environmental constraint is a restriction on a physical (human/device such as sensor) object’s ability to act, perform, construct, or do anything else. In relation to USSNs, environmental constraints that need to be taken into account include the salinity and depth at which an underwater sensor should be positioned for optimal performance. Failure to do so could have a negative impact on the performance of the entire SSN and result in the improper underwater deployment of all SSN components, including servers, sensors, cables, and other physical and telecommunication components [6,7].

**Figure 1:**
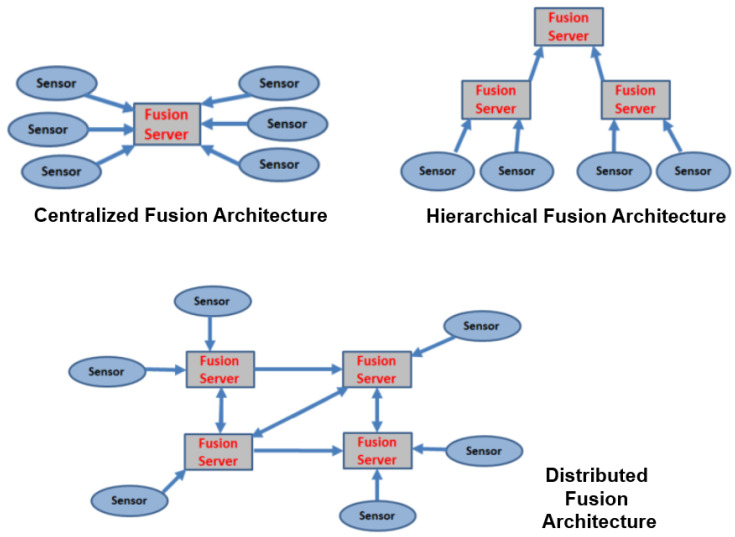
Centralized, hierarchical, and distributed fusion architecture.

USSNs are composed of artificial intelligence (AI) and several specialized sensors [8]. This demonstrates how Smart Sensors are connected to various Smart Fusion Servers with AI Databases [9] through the use of Distributed Fusion Architecture (Figure 1) [10] to carry out multiple tasks like collecting, processing, and storing data continuously and permanently from various sensors dispersed throughout various locations within the same network. Therefore, the primary job of SSNs is to gather environmental data and transmit them to a centralized location or processing system, such a Smart Fusion Server, for evaluation and decision-making.

By now, it ought to be obvious how challenging it is to create USSNs since various resources contribute to its intricacy. Nonetheless, in this article, we specifically discuss the following [7,11,12]: since a distributed system’s architecture consists of many heterogeneous components, it is a complicated system by itself. This indicates that it will be challenging to analyze, design, and deploy USSNs. Certainly, you can expand upon the importance of the design of the USSN and the potential consequences of design mistakes throughout the deployment phase of any USSN project. This is due to the fact that, in the USSN life cycle, the design phase is performed first, coming before the deployment phase.

**Figure 2:**
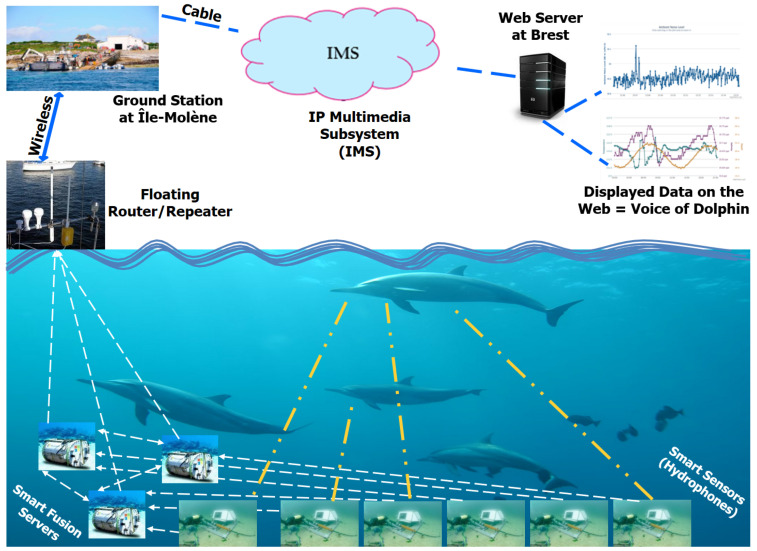
MeDON’s run-time (based on Distributed Fusion Architecture (Figure 1)—an example: N = 6 Smart Sensors and Y = 3 Smart Fusions Servers).

We focus our research on the applications for the Marine Observatories (MOs), which have a common concept but employ diverse technologies. More specifically, our research’s focus is dedicated on the Marine e-Data Observatory Network (MeDON) (Figure 2).

The MeDON project [13] relies on USSNs in order to enable continuous ocean observation. MeDON uses various communication protocols (REST, SOAP, and executive ones) to connect its various physical and logical components (e.g., Smart Sensors, Smart Fusion Servers, and Object Localization Algorithms), and together, these sensors gather data, which is subsequently transferred to the assigned workstations where it operates as a complex distributed information system. This indicates that the structure of MeDON [13] is similar to that of the USSN system, meaning that the issues we previously discussed regarding the complexity and challenges encountered during the USSN project’s design and deployment also arise during MeDON’s design and deployment.

Accordingly, our scope in the MeDON project revolves around the intricate design of the USSN and the associated mechanisms for locating underwater moving objects. This aspect of the project is critical for achieving its overarching goals, which include monitoring and understanding the marine environment. According to [14], the complexity of the design phase is due to: (1) the several expertise domains (information systems, business process, and underlying infrastructure modeling) that the designer(s) must possess in order to model and explain such systems; (2) the MeDON/USSN Information System’s distributed software structure (Figure 2), which is characterized by the fact that individual components, such as Smart Fusion Servers and Smart Sensors, are accountable for meeting specific requirements; and (3) the high levels of accuracy that must be met by the designer(s) for each component that must be deployed underwater throughout the design phase, especially for the physical components such as Smart Sensors. For this purpose, an information system like MeDON or any other USSN has a complicated design phase that demands meticulous attention to detail. In light of this, mistakes/errors committed during this vital phase might have a significant and negative impact on the project’s overall performance [7,11,12].

In terms of our contribution in the MeDON project, we are involved via underwater tracking moving objects. In order to deliver such high-level services, we have to be careful not to make any mistakes during the design phase, especially when it comes to proper and appropriate deployment. Otherwise, this has a negative impact on the overall performance of the MeDON project (e.g., inaccurate object localization in our case). The next section will go into more detail about these detrimental effects.

For this purpose, our main objective is to simplify the work of SSN designers in order to prevent errors during the deployment phase and ensure optimal performance across the whole SSN.

According to [7], architecture (2D or 3D), salinity levels, and operable depth are the essential Underwater Environmental Constraints (UECs) that must be adhered to during the deployment phase of USSNs. Therefore, because of the earlier validation procedure at the design phase, mistakes that may arise during the deployment phase are decreased if we ensure that these UECs are well respected throughout the design phase. Thus, there is a requirement for seamless integration between the MO information system and the communication system (e.g., IMS) [15].

In order to tackle all of the aforementioned issues and to reach our main objective, our research question revolves on improving the SSN’s design phase and streamlining the intricate details of the deployment phase.

**Figure 3:**
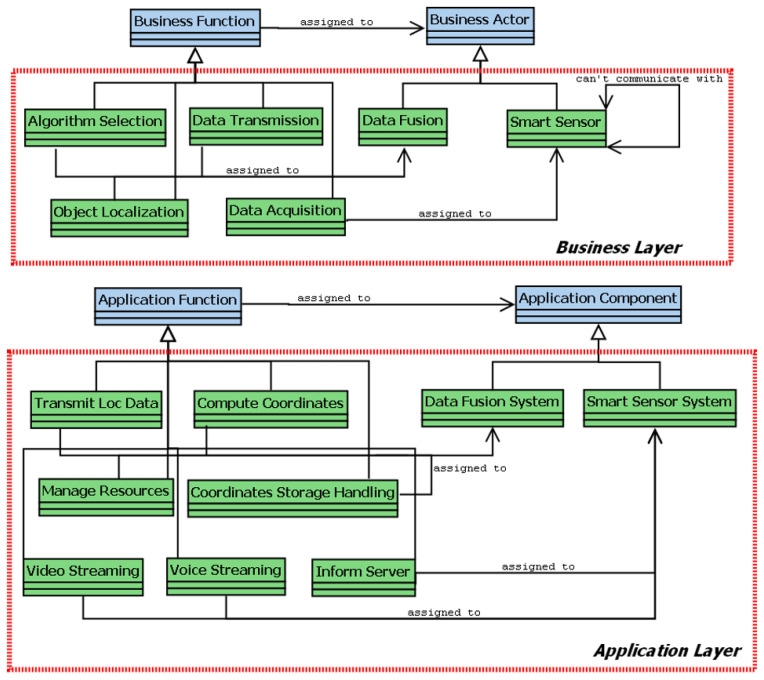
ArchiMO meta-model—extended business and application layers of Archimate.

**Figure 4:**
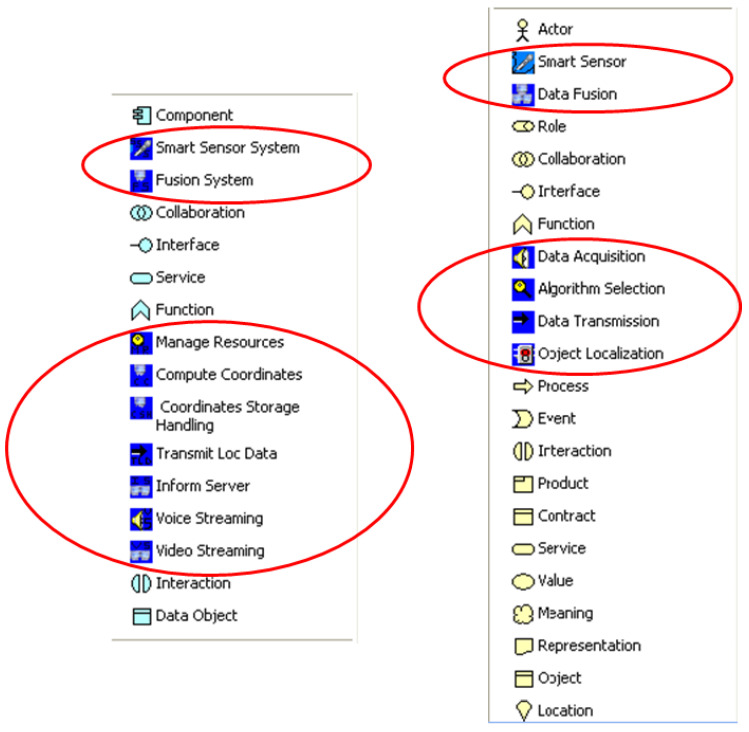
ArchiMO design tool—extended MO business and application layers (palettes) [19]. The MO elements that are added to the pre-existing Archi Tool elements are shown in red circles.

We present an extension to the ArchiMO meta-model and design tool (Figure 3 and Figure 4) in this work. ArchiMO was previously developed and published in [2,3,16]. The ArchiMO meta-model enables us to generate a specific design tool that is coherent with the Archi tool [17] but contains additional concepts, elements, constraints, and relations that are specific to the MeDON/MO domain, and for data fusion concepts [10], we use the ArchiMO design tool. In order to create this generation, we have extended the Eclipse Modeling Framework (EMF) based on the fundamental principles of Model-Driven Engineering (MDE) [18]. EMF is based on ArchiMate, an Enterprise Architecture modeling language consisting of three layers: application, business, and technology. Briefly, the generated design tool ArchiMO helps the SSN designers to model the USSN system and avoid syntax errors that may be made during the design activity.

However, an AI Database including the newly proposed UEC is absent from ArchiMO. As a result, the SSN designer was able to define an underwater sensor during the design phase without the ArchiMO tool preventing him or even warning him about potential mistakes and without informing him about the appropriate UEC values that he should uphold to prevent mistakes during the implementation phase.

In this article, we have extended the ArchiMO meta-model (abstract syntax), concrete syntax, and the design tool by incorporating new Underwater Environmental Constraints (UECs) [7]. Our extension’s approach is based on the utilization of Domain-Specific Modeling Languages (DSMLs). The extended ArchiMO represents the specificity of the SSN domain in terms of certain UECs that are required for the proper deployment of USSNs to ensure its appropriate performance and functioning. Regarding the generation process of a new version for our ArchiMO, it is similar to what we conducted in our previous research in [2,3,16], which will be discussed in the contribution section.

In addition, we have connected our extended ArchiMO design tool with the IP Multimedia Subsystem (IMS) meta-model serves to seamlessly integrate the various Smart Sensors and Smart Fusion Servers within the sensor network with the broader information system via the core network [20,21]. Subsequently, we apply our design model to a model compiler, which generates a simulation code that can be executed directly within the NS-3 network simulator.

The article content is organized as follows: in Section 2, we present the related work that is connected to the design tools. Section 3 presents MO project. In Section 4, we present MDE fundamentals and the DSML, ArchiMate, and AI Databases. Section 5 explains the abstract syntax, concrete syntax, semantics, and AI Database of the proposed DSML including UEC. In Section 6, we present the newly added UEC along with how it is generated with the ArchiMO design tool, as well as the simulation approach. Then, in Section 7, we conclude and discuss our future work.

## 2. Related Work

We provide the relevant work in relation to the design tools in this section. We are interested in the following concerns, which we will define and examine in this section in relation to the concept of Architectural Description Languages (ADLs) [22,23,24,25] and their design tools: (C1) utilizing a language’s syntax or language structure to prevent errors at the design level; (C2) various points of view (a viewpoint is a work product establishing the conventions for the construction, interpretation, and use of architecture views to frame specific system concerns) that are reflected in the architectural description [26]; (C3) design tool extensibility; (C4) the variability of components; and (C5) a platform for execution and testing.

Regarding the concern of preventing errors, the expanded design tool works to stop problems before they happen, saving the designer the trouble of fixing them later. The sources for this error prevention strategy include [27,28,29]. Similar to our methodology, it is circumvented by utilizing the abstract syntax (our suggested concepts), in which we have established and included our particular concepts, constraints, and relations.

Regarding the concern of many viewpoints, the extended design tool offers the designers a variety of viewpoints based on their domains of experience. The design tool in [27,28,29] offers only one viewpoint to suit software development activities. It is not possible for various designers to share a design created with this design tool. This is because there is no architectural framework that produces a design tool that complies completely with the aforementioned ooncer [30]. Our approach takes this into account because of the several EA standard layers that distinguish between different points of view.

As for the extensibility concern, adding new concepts and restrictions to an existing design tool is made possible through the extension of a meta-model [27,28]. As demonstrated in [31,32], our method achieves this by adding additional constraints to the ArchiMate meta-model and creating a new design tool that incorporates these constraints.

Regarding the heterogeneity concern, which is the presence of various components and communications associated with various activities and contexts, in [27,28,29], we encounter this heterogeneity in the software components and models. The diversity of components in our approach is seen in our MO model, which has several Smart Sensors linked to numerous Data Fusion Servers.

Concerning the platform for the execution test, we may find an integration between two distinct platforms, as shown in [33], to provide an automated execution test of a given complex model. Additionally, as shown in [27,28,29], there are platforms on which the designer is unable to test and validate his models or instances. Nevertheless, as per [31], our approach enables us to verify the generated models on an executable platform that is integrated inside the same framework that facilitates model construction (see to Section 6). For instance, messages can be sent and received between Smart Sensors and Fusion Servers using the IMS.

Actually, we should evaluate the detrimental effects of mistakes or sub-optimal sensor network designs on the overall performance of USSNs in order to further elucidate C1. According to C1, these mistakes should be avoided since they may arise from the following outcomes [7,11,12]: (1) Inaccurate and incomplete data collection: this undermines the project’s ability to gather reliable information about the underwater environment, including the quality of services provided such as the movement patterns of marine mammals/underwater moving objects and the state of the ecosystem. (2) Operational inefficiencies: this can introduce an increased energy consumption, high maintenance costs, and reduced sensor network lifespan. These inefficiencies can strain project resources and hinder its long-term sustainability. (3) Data misinterpretation and misalignment with project objectives: this involves drawing incorrect conclusions or failing to identify critical environmental trends, hindering scientific understanding. (4) Wasted resources in terms of designers and analysts: this is costly due to the expenses for redesign and retrofitting. This not only drains project resources but also leads to delays in deployment, potentially impacting the project’s ability to meet deadlines and objectives. (5) Project reputation: this may make the project less attractive to collaborators, stakeholders, and funding agencies. (6) Inappropriate/inaccurate deployment: this could result in redeploying all or some of the SSN’s components. This is costly due to the substantial expenses for the procurement of specialized boats, maritime cables, hydrophones, Smart Data Fusion Servers, and the engagement of diving experts.

Accordingly, certain Underwater Environmental Constraints (UECs) must be considered while deploying USSNs in order to prevent errors that may arise during the design and deployment phases, as we have found in [6,7]. Otherwise, there will be a detrimental effect on the project’s overall performance due to these intricacies, which will increase the likelihood of errors during the deployment phase. Furthermore, it has a detrimental impact on sensor algorithm performance, diminishing its optimization. For example, Smart Sensors (Smart Hydrophones in our case) may only detect raw data and then transfer it to other devices (Smart Fusion Servers in our case) within the same networks without any analysis. This may occur when underwater sensors are not deployed in the proper location (e.g., appropriate operable depth). In this case, the sensor may detect and transmit erroneous and useless data to other devices because sensors function differently depending on the operable depth. For instance, it may lead to incorrect object location detection [34,35]. It is crucial to recognize the risks that will inevitably present during the deployment phase as a result of design phase mistakes.

## 3. Marine Observatories

An analog or digital transducer paired with a CPU and a communication interface is called a Smart Sensor. It is composed of a controller or processor that supports some intelligence, a transducer element, and an electrical signal conditioning system all combined into one package [36]. This type of sensor is known as a system-on-a-chip (SoC) because it combines electronics and a transducer (which changes form) element onto a single silicon chip. The primary goal of combining electronics and sensors is to create an integrated sensor, often known as a Smart Sensor.

In the context of the Marine e-Data Observatory Network (MeDON) project (Figure 2), Smart Sensors should be employed in the project’s development and deployment.

Many advantages come from integrating a Smart Sensor into a MeDON project; here are a few of them: (1) The sensor’s input is processed by the embedded CPU to produce meaningful information. This indicates that the device’s Multiple Controller Unit (MCU) can compute the input data without using up all of its energy. By doing this, the device’s power consumption from the base sensors is reduced. (2) The real-time data gathered by these sensors can be instantly linked to other devices and transferred via an Application Programming Interface (API) without the need for time-consuming intermediate processes. (3) It may receive input by recognizing different data parameters from several information sources and then utilize built-in routines to determine a particular combination of inputs before sending the data to networks that are currently active. (4) It makes it possible for information to be collected automatically, which reduces false noise that is recorded alongside accurate information.

Consequently, a Smart Sensor can gather environmental data more precisely and with lower false noise [1]. In the context of Internet of Things (IoT) technologies (like MeDON), Sensors and Smart Sensors are essential components. Fundamentally, sensors aid in the gathering and processing of data used by IoT devices. In the IoT domain, a Smart sensor can then make certain decisions.

Future big data collecting systems will be developed using Underwater Smart Sensor Networks with a focus on environmental data acquisition [13,37,38]. They enable the interchange and processing of data between the various devices (e.g., Smart Fusion Servers, Smart Sensors). We can install software components on all of these devices to handle and store the data and information. As long as the network is functioning properly, these components can provide new features or services such as the localization of marine mammals or underwater moving objects as the Marine e-Data Observatory Network (MeDON) project (Figure 2), which provides an example of a Marine Observatories (MOs). To provide such services, SSNs ought to consist of a network of specialized heterogeneous devices and communications infrastructure that can record and monitor data at different locations with varying levels of computational and communication capabilities with different communication protocols.

In this situation, to provide such a localization service, the designer should be able to integrate N acoustic Smart Sensors (Smart Hydrophones) connected to Y Smart Fusion Servers to allow for data interchange between them. This exchange’s scenario is predicated on the concept of Distributed Fusion Architecture (DFA) [10] (Figure 1). These servers process the acoustic data that the Smart Hydrophones collected before disseminating it over the network. The same database is used by all servers to store data to be analyzed to become information. The web server, where a web application is configured, receives the processed and analyzed data from the databases servers. As a result, the web server transmits, via a graphical user interface (Figure 2), the information picked up by the hydrophones, such as the voice of the dolphin, to the web clients.

**Figure 5:**
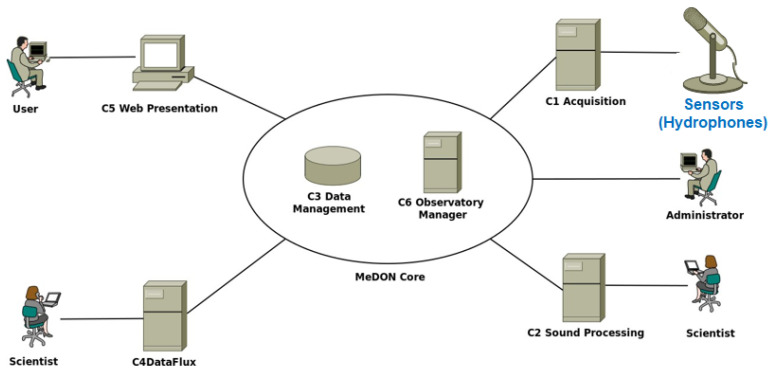
Structure of the distributed software of the Information System of MeDON.

**Figure 6:**
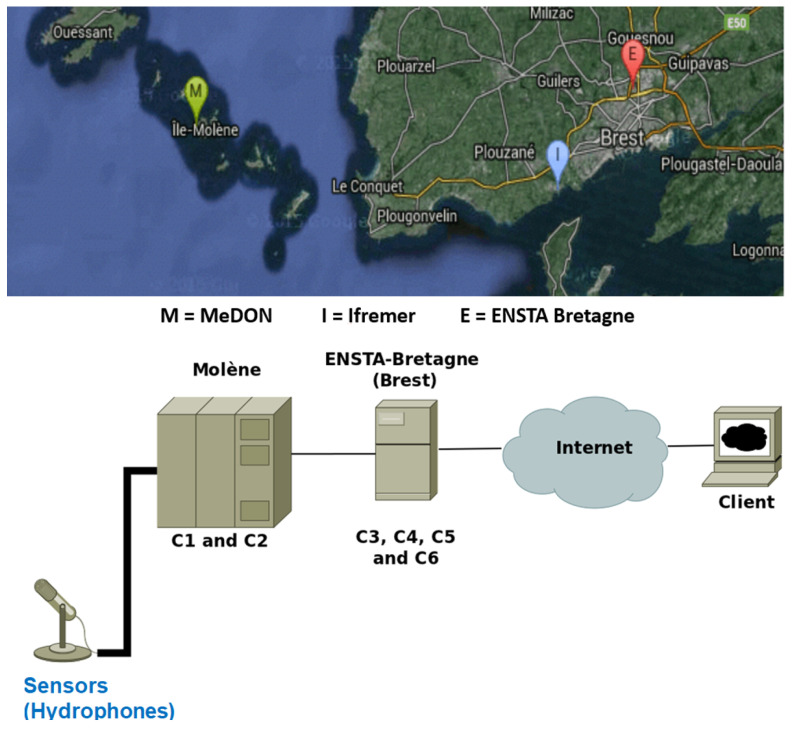
Physical deployment of the Information System of MeDON.

This implementation gives priority to modularity in its architecture. One can develop and deploy the application across numerous sites more easily when it is modular. This is required as we are working with distributed systems [10] (Figure 1). In order to achieve the requested modularity, we choose to break the system down into several components. There are two groups of components (Figure 5): (1) The core components and (2) the functional components. The core components provide the data flow (C6 Observatory Manager), the found data, and part of the stored data functionality (C3 Data Management). The core components should be deployed before all other components. Moreover, the component C6 Observatory Manager should be the first one to be deployed. Then, it is possible to deploy the component C3 Data Management. The other components can then be deployed. Furthermore, as depicted in (Figure 6), MeDON has been physically deployed in many places by connecting the various components using SOAP Web Services.

The terms “hydro” (water) and “phone” (sound) combine to form the word hydrophone. To put it simply, a hydrophone is an underwater microphone that is used to measure sound in the water [39].

Acoustic waves are considered the most efficient carriers for underwater applications and long-distance information transmission (e.g., MeDON) due to their significantly longer propagation distances in water compared to electromagnetic waves. Additionally, because of issues with multipath propagation, time fluctuations in the communication channel, short bandwidth, and the need for powerful signals, the usage of electromagnetic wave-based communications is severely restricted underwater. For this reason, sound has been applied extensively in the undersea field up to this point [39].

In the field of underwater acoustic measurement, several hydrophone structures with various operation mechanisms are employed to fulfill the various needs of the circumstances. These gadgets include underwater military weaponry and SONAR (Sound Navigation and Ranging) equipment, in addition to standard commercial electronics.

A 2D or 3D array/network of hydrophones can be utilized in place of a single hydrophone, depending on certain approaches and algorithms, to enhance performance and provide additional features in both active and passive modes.

**Figure 7:**
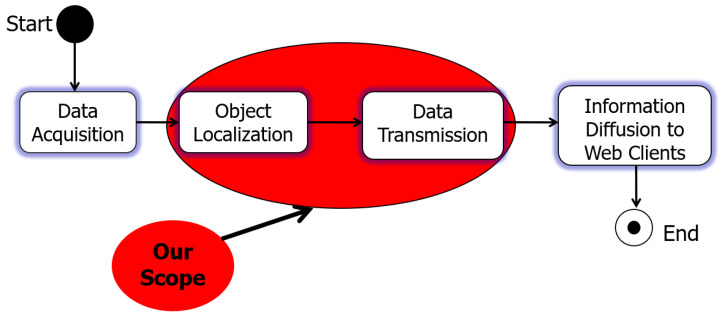
Logical activities of MO/MeDON.

More specifically, the logical sequential activities of MeDON are represented by (Figure 7). Our article focuses on the two activities (Object Localization and Data Transmission Activities) that are denoted by the red circle in (Figure 7).

## 4. Model-Driven Engineering (MDE), Domain-Specific Modeling Languages (DSML), and Artificial Intelligence (AI) Databases

They are broken down into three sections:

### 4.1. Model-Driven Engineering (MDE)

MDE [26] is a software development method that focuses on creating and exploiting domain models. It enables the use of models for simulating, estimation, understanding, and communication. The modeling idea and model transformations in MDE aid in managing complexity. Modeling aids in the high-level abstraction of the design, and model transformation aids in the generation of design tools.

In our approach, modeling tools follow the constraints and represent the concepts that are defined in the meta-model (the meta-model defines by itself a language for describing a specific domain of interest [40]). Similar to programming languages, it makes it possible to instantiate lots of conforming models [41]; numerous programs can be implemented relying on a specific programming language (e.g., C, C++, Java, etc.).

The modeling and meta-modeling processes are made easier by the powerful environment offered by the Eclipse IDE, which also supports a wide range of model transformation languages.

Model transformations enable the direct and automatic generation of design tools and simulation programs while taking into account meta-models and model instances. Each model transformation is based on a set of guidelines that define and regulate the transformation procedure. Models that adhere to various meta-models may be mapped by the transformation rules (on the same abstraction level) such as ATL [42], or map between different domains using one meta-model for the source model to generate texts/codes (e.g., XPAND [43]).

In our case (Figure 3), the input model reflects the design at a very high level of abstraction, and the meta-model (the extended ArchiMate meta-model) represents the abstract syntax [26,44]. Our automated code generation approach connects the simulation scripts and the design model directly [45]. As a result, it minimizes implementation errors and shortens the implementation time for complicated simulation programs.

### 4.2. Domain-Specific Modeling Languages (DSML)

According to [46], Domain-Specific Modeling Languages (DSMLs) allow designers from various fields and backgrounds to take part in software development tasks and to express their own requirements using domain concepts. The three parts of a DSML [47] are semantics, abstract syntax, and concrete syntax. The relationship between modeling concepts is defined by the abstract syntax.

Concrete syntax comes in a variety of forms, including visual, textual, XML-based, etc. [48]. The representation of the abstract syntax is defined by a set of rules that are connected to the concrete syntax. Semantics, which are connected to abstract syntax, define a model’s meaning. They serve as well-defined model rules that limit the use of the concrete syntax [47]. According to [48], modeling languages are used to describe systems with a high level of abstraction. We define distributed systems for MeDON/MO in connection to our goals. UML only has one layer that encompasses all of the design concepts, and these concepts are too broad to meet our needs. For further information, see Horton [49]. Because it relies on the TOGAF framework and can describe systems from the IT domain and share multiple points of view during the design process, ArchiMate is the modeling language we chose [26]. Additionally, as of January 2018, the ArchiMate meta-model by The Open Group can be used to generate the most recent iteration of the NATO Architecture Framework (NAF v4) [30]. A standard for creating architectures is NAFv4.

The Enterprise Architecture (EA) framework is a fundamental component of ArchiMate [26,50]. The system design is broken down into the business, application, and technological layers. According to our methodology, we show these levels as follows:

1. Business Layer: describes the roles and responsibilities of the end user. It explains how the end-user views the service operations and how they flow together.
2. Application Layer: describes the features and software parts of the service. It explains the capabilities and method of operation of the system being studied.
3. Technology Layer: describes the underlying platform’s hardware components, topology, signaling protocols, and functions. It provides information about the execution platform’s functionalities that the application layer’s functions can utilize.

### 4.3. Artificial Intelligence (AI) Databases

AI databases are designed to handle three different types of data: unstructured, semi-structured, and structured. All of these data types are necessary for creating and utilizing AI models.

The many formats in which information is saved and structured are referred to as “types of data” when discussing AI databases. These formats are crucial for processing, analyzing, and using data to create AI models [9].

Structured data are set up in a very clear and organized way. Each data entry contains particular fields and features with clearly defined data types, and the system follows a transparent data model.

Tables, spreadsheets, and classic relational databases all contain organized data as examples. The links between the data elements are well specified in structured data, which makes them simple to query and analyze with established techniques. Structured data for AI applications might include numbers, categorized labels, dates, and other clearly specified information.

## 5. Contribution

Domain specificity (MO) in our situation is represented by concepts/operations and constraints that are generally represented by a meta-model of Domain-Specific Language (DSL) [48]. Information systems are modeled and described using a modeling language that is provided by a meta-model. It includes the language’s abstract syntax, which explains its constraints in terms of the concrete syntax that the design tool can use.

Two views comprise our previous proposed ArchiMo meta-model ([16] (Figure 3): one for the application layer and another for the business layer. In order to connect the information system with the core network at the technological layer, we rely on a meta-model for IMS that offers an underlying platform in [16].

In this section, we present our contribution to extend the ArchiMO meta-model that we previously developed and published in [2,3,16]. Our previous contribution ArchiMO is presented in (Figure 3 and Figure 4).

The ArchiMO meta-model represents the domain specifications of MO. It enables us to generate a specific design tool that is coherent with Archi tool [17] but contains additional concepts, elements, constraints, and relations that are specific to the MeDON/MO domain and for data fusion concepts [10]. Briefly, the generated design tool ArchiMO helps the SSN designers to model information systems such as MeDON and avoid syntax errors that may be made during the design activity.

Our primary goal in writing this article is to improve ArchiMO’s intelligence by increasing its ability to identify mistakes that SSN designers may have made when creating and developing MO models. Additionally, we aim to keep the entire deployed SSN operating at peak performance and avoid any malfunction.

To reach this goal, we have extended the ArchiMO meta-model (abstract syntax), concrete syntax, and the design tool by incorporating new Underwater Environmental Constraints (UECs). This extended ArchiMO represents the specificity of the SSN domain in terms of certain UECs that are required for the proper deployment of USSNs to ensure the appropriate performance and functioning of SSNs [6,7,11,12,34,35]. Additionally, it enables us to generate a new and an updated version of our ArchiMO design tool that is consistent with Archi and includes the new UECs.

Relying on [6,7,34] and according to MeDON project [13] (Figure 2), SSN information systems must meet certain UEC requirements in order to be deployed properly. This implies that every physical component of SSNs, including Smart Sensors (SSs) and Smart Fusion Servers (SFSs), must comply with these UECs. Otherwise, inadequate service quality on each deployed component level (e.g., inaccurate/useless detected data by the underwater SS) and overall SSN performance (e.g., erroneous/useless gathered, analyzed, and treated data that are provided by underwater SFSs after receiving data from different underwater SSs) may result from this. As a result, we identify the following UECs that are interrelated, depend on each other, and need to be taken into account when deploying underwater SSSs/SFss to ensure the appropriate performance and functioning of SSN [11]: (1) architecture (2D or 3D); (2) salinity level; and (3) operable depth.

SSN designers need to be aware of the various ways in which these UECs are inter-dependencies and related in terms of values [6] such as the two following scenarios: (1) If the experts want to deal with 3D architecture, they should be aware of the salinity level (e.g., sea or shallow water) at which they wish to install the physical components of the USSNs (e.g., SSSs/SFss). The deployment of SSs/SFs should be carried out at a depth of 10,920 m underwater if the salinity level is sea. However, the deployment of SSs/SFs should be carried out at a depth of 3000 m underwater if the salinity level is shallow water. (2) If the experts want to deal with sea salinity level, they should be aware of architecture type (e.g., 2D or 3D) of which they wish to install the physical components of the USSNs. The deployment of SSs/SFs should be carried out at a depth of 110 m underwater if the architecture is 2D. However, the deployment of SSs/SFs should be carried out at a depth of 10,920 m underwater if the architecture is 3D.

**Figure 8:**
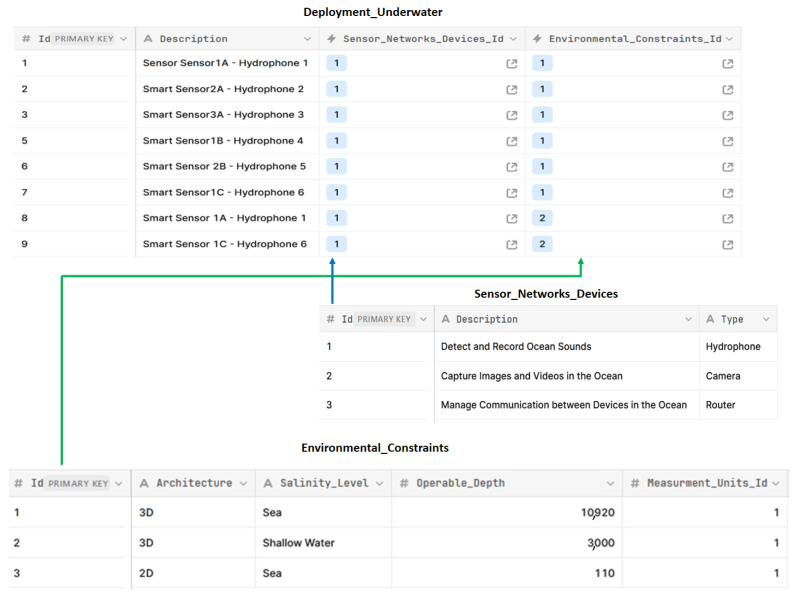
Operational AI database model—underwater environmental constraints.

In this article, we have extended our ArchiMO meta-model by incorporating new UECs. In practice, we have specialized the definition of the Smart Sensors according to the UECs. This specialization involves creating an AI Database (Figure 8) that is connected to the Eclipse Modeling Framework (EMF) by implementing java code in the EMF (e.g., JDBC connection string, etc.), as well as by extending the Eclipse Modeling Framework (EMF), which is based on the ArchiMate and ArchiMO meta-models, by implementing Java code (e.g., control instructions such as if, else, etc.).

In technical terms, the extension of ArchiMO is carried out by: (1) using the ArchiMate modeling language together with learning control (which is one application of Artificial Intelligence) that is based on an AI Database and includes structured data that are necessary for designing and deploying AI models in order to properly model USSN systems [9]. AI Database, a relational database, is created containing various related entities that describe how the data of the proposed UECs could be inserted, modified, and retrieved in an organized and structured manner using a relational database management system (MySQL) based on Structured Query Language (SQL); (2) implementing Java code in the EMF (e.g., JDBC connection string, etc.), in order to invoke the values of UEC during the design activity.

**Figure 9:**
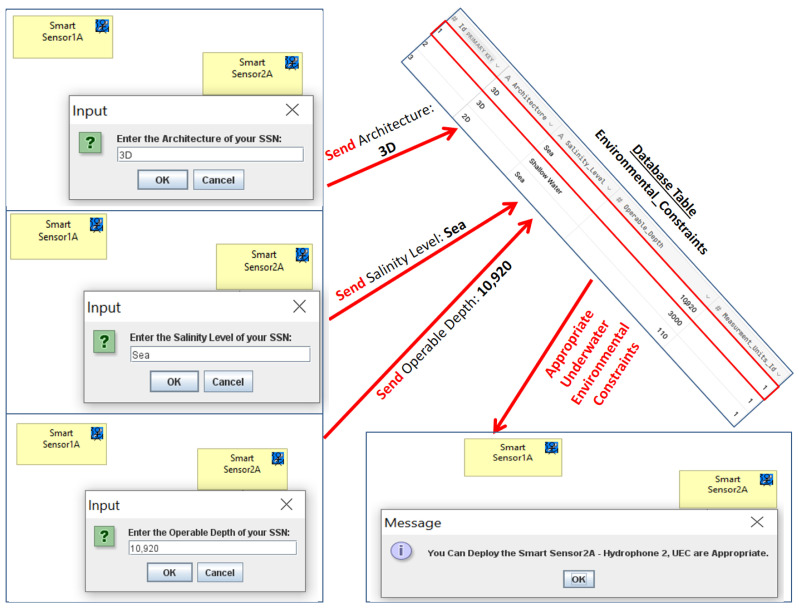
Appropriate underwater environmental constraints.

**Figure 10:**
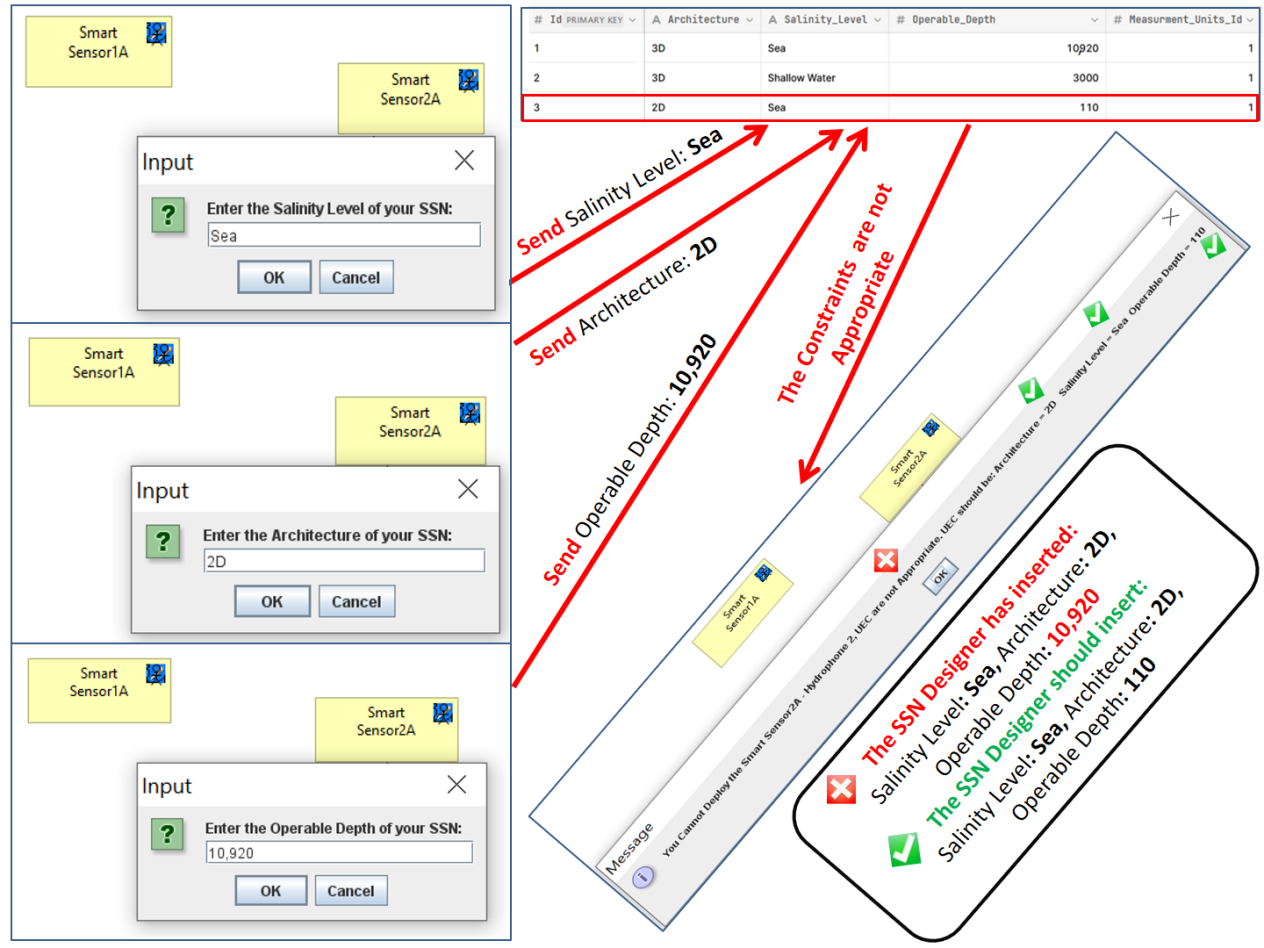
Inappropriate underwater environmental constraints.

Then, in accordance with Archi, we have generated an upgraded version of our ArchiMO design tool (Figure 4) that incorporates the new proposed UEC. Additionally, when using this tool, the built and implemented AI Database is invoked by the implemented Java code in order to retrieve data (Figure 8) related to UEC that must be respected by SSN designers (Figure 9 and Figure 10). This implementation is the grammar of the new proposed DSML.

To visualize and have a graphical view for the added UECs, we utilized the generated version of ArchiMO during the creation of an MO model. This design tool helps the designer to model the information system in a highly abstract way by dragging and dropping the elements (e.g., Smart Sensors) and relations from the palette. The potential to employ the newly added UECs and their proper operation is verified during model editing in the following way: when a designer taps the SS icon in the ArchiMO palette, our extended ArchiMO design tool uses the AI Database to extract the relevant UEC’ values and the targeted SS’s accompanying constraints according to a valid primary key that should be assigned by the designers. At this stage, ArchiMO continues asking the SSN designer to enter the right primary key of the targeted SS and then the right architecture, salinity level, and operable depth in order to compare and verify the entered values of UEC with the retrieved values from the AI Database. At this point, if the SSN designer executes one of the next two scenarios, he will obtain confirmation from the framework that he can deploy the Smart Sensor (Figure 9): (architecture: 3D, salinity level: sea, and operable depth: 10,920) or (architecture: 2D, salinity level: sea, and operable depth: 110). In the opposite scenario, as seen in (Figure 10), the SSN designer will be informed that he is unable to deploy the Smart Sensor due to one or more improper UEC. Consequently, these constraints prevent the SSN designers from inputting the improper UEC for SSs/SFs/Fixed Nodes.

Our contribution replies to the concerns that we have presented in section II by: (C1) Enabling the SSN designers to cease entering the incorrect UEC for SS (Figure 9 and Figure 10), ArchiMO helps to avoid potential syntax errors during the design activity. (C2) The ArchiMO design tool considers different domains of experience; each domain expert works in a specific layer (business, application, or technology) as the model created in section VI. It provides three layers according to each domain specificity. One notable characteristic of Enterprise Architecture (EA) frameworks is their ability to facilitate the sharing of multiple viewpoints [51]. This, in turn, simplifies the complexity of individual views, making them more manageable. The Enterprise Architecture frameworks also introduce interoperability challenges when attempting to integrate various viewpoints with their respective dedicated software. (C3) We have expanded the Eclipse Modeling Framework (EMF), an open and standard framework based on the ArchiMate and ArchiMO meta-models, by implementing java code and building an AI database that is invoked. Subsequently, we have generated an upgraded version of our design tool (Figure 4) to have a specific one like ArchiMO that incorporates the new proposed UEC. (C4) The ArchiMO design tool provides the ability to deploy SSN models (the model created in section VI) that contains heterogeneous components in terms of software; hardware; functionality; and communication protocols such as Smart Sensors, Smart Fusion Servers, and Localization Algorithms.

## 6. Underwater Object Localization Service Case Study Relying on Multi-Sensor Data Fusion

The data fusion analysis theory [10] is utilized in this case study to locate things, and it primarily demonstrates how we apply our method to combine underwater sensor networks with the information system employing IP technology and IMS. We use the localization service as an illustration of one of these uses. The underwater moving object localization service seeks to pinpoint an object’s location following its detection by a sensor or group of underwater sensors. For underwater applications where electromagnetic waves cannot travel great distances, acoustic hydrophones are suitable sensors. The topology of the Fusion Servers and sensor network in the context of data fusion is represented by information graphs (centralized and distributed architecture). According to [10], information graphs (Figure 1) provide “convenient means to understand how fusion process flows impact a network system”. To offer a thorough and comprehensive picture of an environment or process of interest, data fusion [52] integrates information from sets of disparate sources. Sensors [53] are the sources of data used in the MeDON project. These data are then merged using multi-data fusion techniques, as described in [10]. The target location is determined and updated by the localization algorithms applied in the server (Figure 1) node (e.g., fusion node).

Our newly proposed UECs, which are introduced in this article, were validated by using the most recent version of the ArchiMO design tool, which includes the UECs and the previously added MO concepts. We built a model that localizes any underwater moving object (in our case, MeDON).

In order to validate this, the newly added UECs and their appropriate functioning are being used in the following manner while creating a MO model: upon tapping the SS icon inside the ArchiMO palette, our extended ArchiMO design tool leverages the AI Database to retrieve pertinent UEC values and the associated constraints of the selected SS, contingent upon a legitimate primary key that the designers must designate. In order to check and compare the input UEC values with the retrieved values from the AI Database, ArchiMO now asks the SSN designer to enter the correct architecture, salinity level, and operational depth for the intended SS. The SSN designer will now receive confirmation from the framework that he can deploy the Smart Sensor if he performs one of the following two scenarios (Figure 9): (Architecture: 3D, Salinity Level: Sea, Operable Depth: 10,920) or (Architecture: 2D, Salinity Level: Sea, Operable Depth: 110). In contrast, the SSN designer will be notified that one or more incorrect UEC prevents him from deploying the Smart Sensor (Figure 10). As a result, these constraints stop the SSN designers from entering an incorrect UEC for Fixed Nodes, SS, or SFS.

The defined model is then used to run various error checks and automatically produce simulation code for NS-3 using a model compiler (see to Section 6.4) that we have created in [2,3], and [21]. A standard and classical networking simulator, the NS-3 tool, is used to run this simulation code.

**Figure 11:**
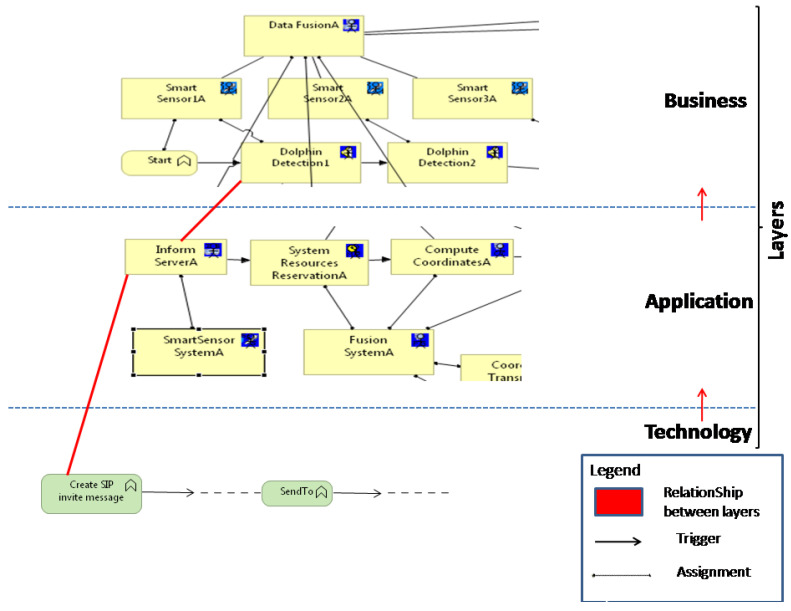
Consistency between business, application, and technology layers [2,3].

The design model comprises three views pertaining to the ArchiMate layers (Figure 11): technology, application, and business. To guarantee interoperability across levels, this figure demonstrates the use of certain relationships like “Used by” and “Realization” to associate the many produced models according to the various ArchiMate layers. By utilizing the inter-relationships (shown by the red lines in Figure 11) among these layers, the various designers involved can combine their separately produced business, application, and technology models into a single, coherent MO model.

### 6.1. Business Model

Figure 9 and Figure 10 illustrate how the elements and constraints (like UEC) found in the proposed meta-model and ArchiMO design tool can be used to define the business model at the time of creating each Smart Sensor (hydrophone). In the context of MO, the object localization system seeks to find and identify an object as it comes into the range of one or more sensors. Sensors are connected to Fusion Servers, which employ a distributed algorithm to determine the object’s position based on the data collected from these sensors.

In our approach, we concentrate on the communication among the several nodes that comprise the MO model. It is possible to add internal actions to the simulator’s modules. Our goal is to demonstrate our capacity to model the MO scenario using the IMS core-network and to produce simulation codes that can be used in NS-3 directly. This aids in assessing the design according to the networking concepts and the constraints that are defined in the meta-model (DSML).

The various tasks and operations that must be carried out while localizing an object in an underwater environment are represented by the business model. Six Smart Sensors and three Fusion Servers are assumed to be part of the system’s architecture. In the network design, both the fusion server and the Smart Sensor are regarded as end-user terminals even though they are crucial components of the information system. As a result, we decide to depict their roles in the business and application layers, leaving the technical layer to depict the network architecture and roles associated with IMS communication exchanges.

Typically, each Smart Sensor finds the dolphin on its own and relays the information to the fusion server, which connects to the other sensors. After merging these inputs, the fusion server employs a localization algorithm to determine the dolphin’s specific location underwater.

**Figure 12:**
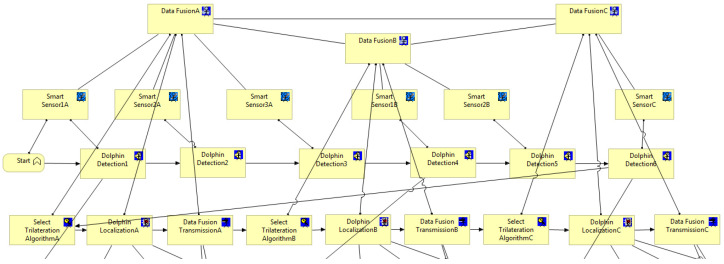
Business view from the underwater object (dolphin) localization model [16]—ArchiMate Business Layer.

The many tasks involved in the item localization process are represented by the business layer of ArchiMate (Figure 12). The dolphin is detected by the several Smart Sensors (1A, 2A, 3A, 1B, 2B, and C) when it passes across the detection region. DolphinDetection is the function that makes this detection possible. Subsequently, each DataFusion center determines the appropriate algorithm for object localization. The DataFusion centers perform this process (trilateration), after which the data are transmitted via the DataFusionTransmission function between the Data Fusion Servers for analysis and subsequent determination of the object’s coordinates.

### 6.2. Application Model

**Figure 13:**
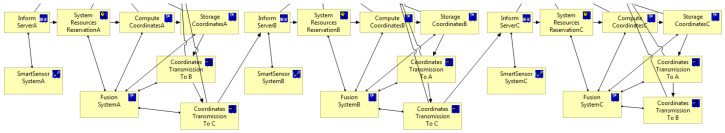
Application view from the underwater object (dolphin) localization model [16]—ArchiMate Application Layer.

The application model is a representation of the system’s functions and application components (in this example, the MeDON information system). The model in (Figure 13) illustrates several associations that connect application and business functions. The significance of the triggering links between two functions is conveyed by these associations. It explains what a call between two functions means. For instance, in accordance with the assignment relationship between functions and components, the SmartSensorSystemA component calls the InformA application function when sensor1A detects a dolphin. The various triggering relationships throughout the application functions then dictate the order of execution. The following two activities, ComputeCoordinatesA and StoreCoordinatesA, are carried out by the FusionSystemA with the resources allocated by the application function SystemResourceReservationA. Next, using the function CoordinatesTransmission to B, the coordination data are transferred from the active DataFusion component to the following DataFusion component, and so on. This process is repeated until the coordinates are determined with greater accuracy while accounting for the detection data gathered by the various Smart Sensors.

### 6.3. Technology Model

**Figure 14:**
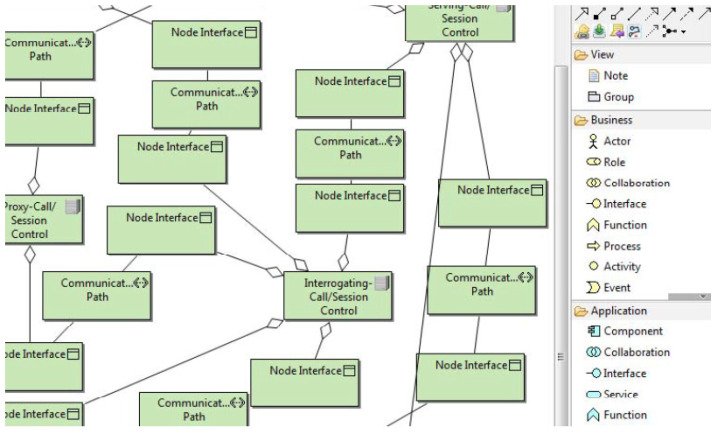
An excerpt of the technology view from the underwater object (dolphin) localization model [16,21]—ArchiMate Technology Layer.

The topology and the tasks to be carried out in each of the network’s nodes are described in the technology model. We concentrate on the technological implementations of the InformA function from the application layer because of its extensive design. An extract from the extensive model (Figure 14) is showcased. To carry out the InformA application function, a wide range of technology functions such as SendTo are connected at the technology layer. A message of type SIP or Diameter can be forwarded or sent from one node to another via the SendTo function.

In the context of technology, we have extended the ArchiMO tool in [21,26] by utilizing the IMS meta-model and ArchiMate. IMS standards are mostly considered and contained in this extension [54]. After the simulation program is executed, the NS-3 simulator creates an animation script. The NetAnim tool imports this script to display the simulation scenario’s animation (see to Section 6.4, which depicts the messages that are transmitted and received between the various nodes. The transparency of our model transformation methods is demonstrated by the produced topology and by watching the message exchanges between the various nodes.

### 6.4. Compilation and Simulation

An XMI file is generated using the enhanced ArchiMO design tool to reflect the graphical design. This facilitates the design model’s interaction with other tools.

**Figure 15:**
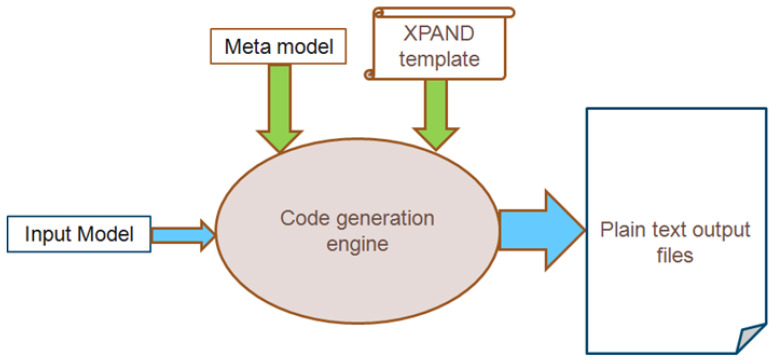
The code generator workflow in XPAND language [2].

The simulation code is generated by our own domain-specific model compiler using the XMI file as an input (Figure 15). This saves a significant amount of development time by concealing from the designer the complexity involved in building simulation programs.

For UEC, the code generator requires the input model generated by the ArchiMO design tool in addition to the meta-model containing the abstract syntax of DSML.

The mapping rules between the model elements and their representations in NS-3 are contained in the XPAND template in (Figure 15) [45]. The code is prone to errors, as evidenced by the compilation and execution results when we ran the resulting code in NS-3 (version 3.13). To analyze the results of the simulation, traces and logs (such as PCAP files) were created.

**Figure 16:**
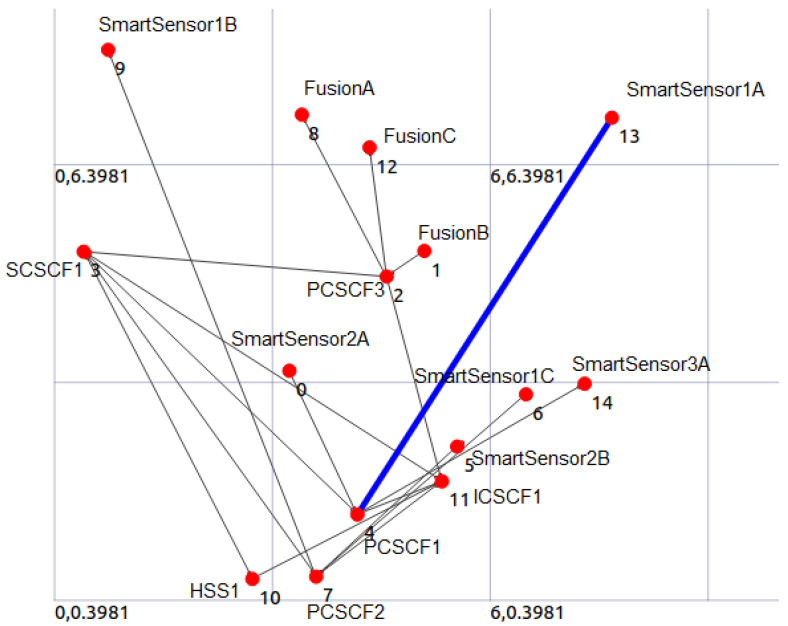
The network topology for the underwater object (dolphin) localization example is represented by a snapshot taken from the NS-3 simulator’s Net Animator tool [2].

The architecture of the system design created by NS-3 for the specified design model is displayed in Figure 16. For each design model element, NS-3 produced a hardware representation (nodes, interfaces, and wires). A message that is transmitted and received at any given time between two nodes is represented by the blue stream. This attests to the behavioral aspects being mapped in the anticipated manner.

This model compiler has an error section in addition to code generation. When used, the defined MO model is used as input to build a simulation code that is ready for simulation. For this reason, we give an illustration of an error that might be found prior to turning the MO model, which was established in the preceding section into a simulation code.

**Figure 17:**
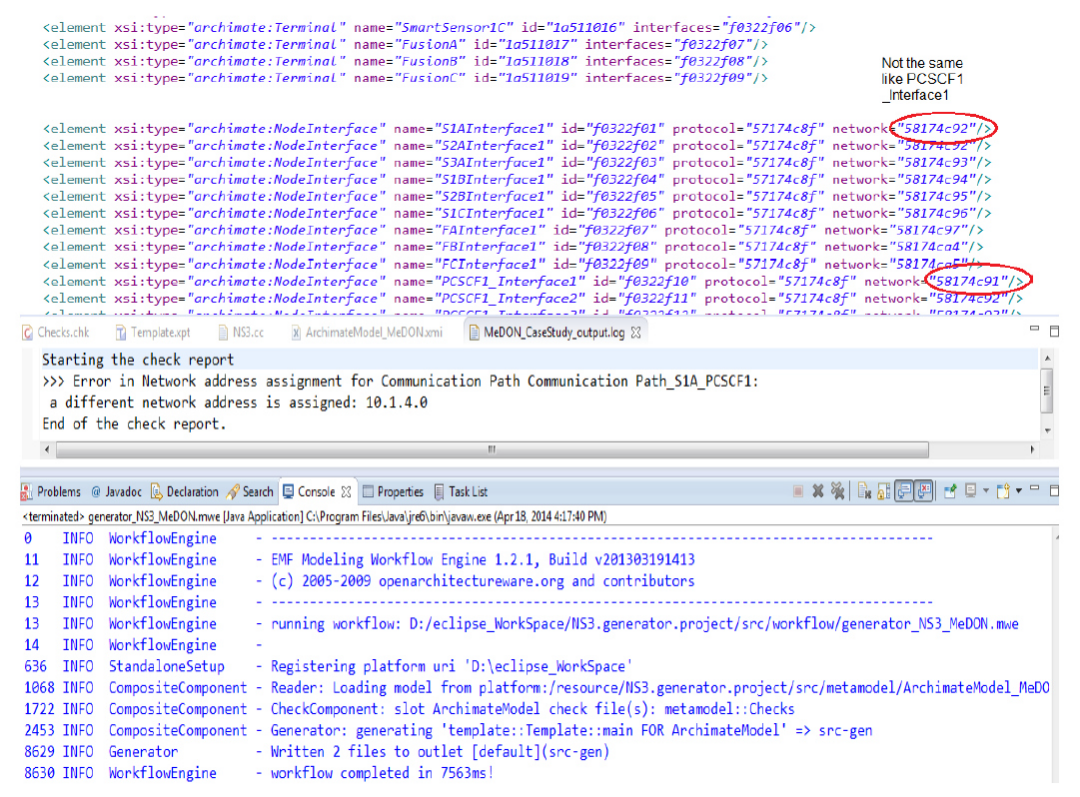
An example of an error detection in design model of Marine Observatory system [16]. The IP addresses of the PCSCF1 IMS node and SmartSensor1A are two different IP addresses shown in the red circles.

To demonstrate the iterative process when we face an error, we have chosen an example that contains an error. We identified a communication failure between the P-CSCF1 IMS node and a Smart Sensor interface. There is a conflicting IP address causing this problem. We change one of the P2P link’s network addresses (Figure 17). A log file including an explanation of this problem is generated by the model compiler (Figure 17). This is where the following question can be posed: How is the model compiler able to identify these errors?

Actually, according to [16], some error detection rules are implemented using the XPAND code generator template. The generating rules that are in charge of creating the simulation situation come before these rules. The procedure in question is accountable for producing a log file that includes an explanation of the problems found in the design model (Figure 17). Since there is a mistake in this figure, we repeat the relevant task until the fault is fixed and a new version of the MO model is created to be simulated. Next, we use the model compiler to iterate the validation task on the updated version, and the outcome is free of architectural design errors. It is challenging to predict the number of iterations required to produce a reliable model, though.

Our approach has been used to several application domains and network simulators (Video Conferencing System [44,45] and MO Context [16]). The underlying platform (IMS), which represents the Platform Specific Model (PSM), is the common design concept shared by all of these use cases [48].

Put differently, by addressing the underlying platform that is represented in the technology layer, we might modify the application domain while still relying on ArchiMate and our extensions (DSML) if we were to use a single tool (such as NS-3). This validates that the models generated by our extended design tool (ArchiMO) adhere to the same meta-model and domain-specific concepts/constraints.

In order to address the problem raised in Section 2, our testing approach does the following: (C5) give the designer the capacity to test and validate his model developed in MO on an executable platform that is part of the same framework in which the designer generates MO. This is achieved by first creating the model’s simulation code and then running it through NS-3.

## 7. Conclusions and Future Work

We have discussed and presented Underwater Environmental Constraints at a high abstract level in this study. These constraints are DSML extensions coupled with the AI Database for the MO context.

We illustrated the proposed UECs and ArchiMO design tool, using a Marine Observatory case study. We presented a defined model for MOs showing their different views: business, application, and technology. These models are created with the help of our extended version of the ArchiMO design tool in terms of abstract syntax, concrete syntax, AI Database, and semantics. This tool includes the newly proposed UEC based on MDE fundamentals. The system model is then validated by simulating the resulting consistent model with the NS-3 network simulator.

Our extended ArchiMO tool protects against design mistakes earlier than traditional design processes/activities and the code generation stage. We rely on a standard and open tool (Archi) that we extend through developing the modeling language and java implementations.

Another benefit of our proposed ArchiMO deign tool is its extensibility. Depending on the development of the SSN domain, the developers may extend it and add new (IIoT)/SSN concepts and constraints, as well as incorporate new and recent useful data into the AI-Developed Database, which is one of the most popular and efficient ways to enhance the efficiency and precision of the AI learning control.

The additional UEC concepts and constraints can be reused in many applications, activities, models, or instances thanks to ArchiMO. Because ArchiMO’s palette includes specific concepts and constraints, it also shortens the time required for the design process. Additionally, we maintain the normative concepts and constraints in the abstract syntax (meta-model) of ArchiMate since the recently added UECs inherit concepts from standard ArchiMate elements.

The opposite is also true: expressing and meta-modeling domain knowledge and enhancing the performance of AI learning control are challenging tasks that demand expertise and a high degree of accuracy, particularly when setting the DSML in accordance with the meta-model requirements and standards.

We will extend our ArchiMO meta-model and design tool by including new Intelligent Internet of Things (IIoT)/Smart Sensor Networks concepts, relationships, and UECs in order to satisfy and cover the most possible required operations, concepts, and activities in the context of SSNs and IIoT. In addition, we will expand the AI-Developed Database by adding more recent and useful data to it.

## Data Availability

Data are contained within the article.
